# Does parent–child agreement vary based on presenting problems? Results from a UK clinical sample

**DOI:** 10.1186/s13034-017-0159-2

**Published:** 2017-04-19

**Authors:** Kalia Cleridou, Praveetha Patalay, Peter Martin

**Affiliations:** 1 Anna Freud National Centre for Children and Families, 12 Maresfield Gardens, London, NW3 5SU UK; 2 Research Department of Clinical, Educational and Health Psychology, University College London, Gower Street, London, WC1E 6BT UK; 3 Institute of Psychology, Health and Society, University of Liverpool, The Waterhouse Building, Dover St, Liverpool, L3 5DA UK

**Keywords:** Parent–child agreement, Internalising, Externalising, Presenting problems

## Abstract

**Background:**

Discrepancies are often found between child and parent reports of child psychopathology, nevertheless the role of the child’s presenting difficulties in relation to these is underexplored. This study investigates whether parent–child agreement on the conduct and emotional scales of the Strengths and Difficulties Questionnaire (SDQ) varied as a result of certain child characteristics, including the child’s presenting problems to clinical services, age and gender.

**Methods:**

The UK-based sample consisted of 16,754 clinical records of children aged 11–17, the majority of which were female (57%) and White (76%). The dataset was provided by the Child Outcomes Research Consortium , which collects outcome measures from child services across the UK. Clinicians reported the child’s presenting difficulties, and parents and children completed the SDQ.

**Results:**

Using correlation analysis, the main findings indicated that agreement varied as a result of the child’s difficulties for reports of conduct problems, and this seemed to be related to the presence or absence of externalising difficulties in the child’s presentation. This was not the case for reports of emotional difficulties. In addition, agreement was higher when reporting problems not consistent with the child’s presentation; for instance, agreement on conduct problems was greater for children presenting with internalising problems. Lastly, the children’s age and gender did not seem to have an impact on agreement.

**Conclusions:**

These findings demonstrate that certain child presenting difficulties, and in particular conduct problems, may be related to informant agreement and need to be considered in clinical practice and research.

*Trial Registration* This study was observational and as such did not require trial registration

## Background

In recent years, increasing emphasis is placed on incorporating perspectives from multiple informants, such as parents, teachers and children, in the way Child and Adolescent Mental Health Services (CAMHS) are delivered and monitored across the UK [1, 2]. Nevertheless, considerable discrepancies are often found between different informants when reporting on the child’s psychopathology, with most studies reporting low to moderate agreement, for a variety of measures and populations [3–6]. For instance, Goodman and colleagues reported varying agreement in a clinic sample between children and parents (mean r = .58), children and teachers (mean r = .39) or parents and teachers (mean r = .39) [6]. Informant discrepancies can pose several challenges for services, as clinicians are often faced with the dilemma of deciding what information they should take into account for assessments and treatment planning [7]. A common reaction is to assume that one informant provides more relevant information than the others and base decisions solely on that person’s report [8].

This can have several consequences for clinical practice, such as rendering it harder to identify the children that are in need of services, to unpick the true level of difficulty for a child or determine treatment efficacy [5, 9–11]. When this leads to the child’s reports being disregarded it poses a threat to the rights of the child and their engagement with the treatment process [12]. Hence, a better understanding of reporter disagreements is relevant not only from a measurement perspective but for also informing clinical practice and research [1, 13–15]. This article will specifically explore the agreement between parents and children on reports of the child’s difficulties.

### Child characteristics influencing parent–child agreement

Most existing literature has explored how agreement varies as a result of the symptom being reported, but not whether this varies as a result of the child’s presentation. One study [16] explored, amongst other things, whether parent–child agreement on a child falling in the clinical range of the SDQ, would vary as a result of the child’s diagnostic category. An interesting finding, as measured by the percentage of children and parents in the sample that agreed on the clinical range, was that highest overall agreement was for those in the depressed (70.2%) or anxious (78.7%) diagnostic category, whereas agreement tended to be lower for those presenting with conduct problems (43.1%). Additionally, in cases of disagreement, it appeared that parents identified the externalising problems more than the internalising ones, when the child did not. Consequently, one possible explanation as to why informant discrepancies occur is that certain child characteristics influence the children’s ability to report their own behaviour. Self-reports can be considered a manifestation of one’s perceptions, since an informant’s report would be routed in their personal experience of a problem, and their own characteristics that might have influenced their interpretation [17]. For example, one factor often associated with the ability to self-report is self-awareness [18]; disorders that bias self-perceptions might lead to inaccurate self-reports and lower parent–child agreement.

#### Externalising problems

Several studies have found that agreement between parents and children was higher when reporting externalising symptoms rather than internalising ones and this has mostly been interpreted to be due to the externalising behaviours being more readily observable by the parent than the internalising difficulties [3, 7, 19]. However, disagreements still remain and children often report less behavioural problems than their parents, which might indicate that the underlying reason for the discrepancies is the child’s limited self-awareness [20, 21]. It has been suggested that externalising disorders are often characterized by the failure to reflect on the self and evaluate one’s own behaviour based on feedback from others [22], resulting in positive biases and impaired self-perceptions [23, 24]. This could have a protective and adaptive function, as an attempt to cope with the difficulties of the disorder [25].

#### Internalising problems

Self-reports are considered particularly important for investigating internalising problems, because these concern internal subjective experiences that might not be observed by others [26, 27]. Indeed, parent–child agreement when reporting emotional difficulties is often lower than for externalising, with children reporting more problems than their parents [19, 28]. One common characteristic of internalising is the distortion of cognition [29–31]. An alternative controversial school of thought introduced the concept of ‘depressive realism’, which can be defined as the propensity of depressed individuals to have more accurate perceptions of reality, while non-depressed people are more likely to exhibit positive biases when evaluating themselves [32–34]. This is consistent with studies such as that of Oland and Shaw [22], which highlighted the key role of self-reflection in the development of internalising disorders and the lack of this in externalising problems.

#### Comorbidity of disorders

Hoza, Murray-Close, Arnold, Hinshaw and Hechtman [35] used a longitudinal design over a 6-year period (assessed at 4 time points) to investigate the link between externalising and internalising problems and limited self-awareness. The findings indicated that children with ADHD 8–13 years old presented with more positively biased self-perceptions about their behaviour relative to reports from teachers across the 6 years, compared to the control group of their healthy peers. Their aggression levels at Times 1 and 2 also significantly predicted positive biases in the perception of their own behaviour at later time points, and at the same time positive biases of behaviour at Time 3 predicted later aggression. One explanation provided by the authors for these findings was the self-protection hypothesis, which suggests that positive biases serve as protection to cope with one’s own deficits [25]. Another important finding by Hoza and colleagues [35] indicated that depressive symptomatology was associated with a reduction of these inflated self-perceptions over time. Therefore, since externalising difficulties were associated with an increase in positive biases and internalising with their reduction, it would be interesting to investigate these biases in the context of comorbidity of difficulties.

#### Other child characteristics

With regards to age, Achenbach and colleagues [3] demonstrated that agreement between parents and children was higher for younger children (mean r = .51) than for adolescents (mean r = .41). The authors suggested that this may be because younger children spend more time with their parents than adolescents do, thus their behaviour is more observable. Similar findings were demonstrated by other studies using samples from the general population demonstrated that agreement between parents and children was higher for younger children (mean r = .51) than for adolescents (mean r = .41). The authors suggested that this may be because younger children spend more time with their parents than adolescents do, thus their behaviour is more observable. Similar findings were demonstrated by other studies using samples from the general population demonstrated that agreement between parents and children was higher for younger children (mean r = .51) than for adolescents (mean r = .41). The authors suggested that this may be because younger children spend more time with their parents than adolescents do, thus their behaviour is more observable. Similar findings were demonstrated by other studies using samples from the general population [36]. However, these results were not replicated when investigating clinical samples [37, 38]. Additionally, the effect of gender on parent–child-agreement has also been examined and like with age the results are inconsistent [37, 38].

### Current study

The overarching goal of this study was to investigate the relationship between certain child characteristics and parent–child agreement. This was divided into two main aims.

1. The first aim was to investigate whether the type of presenting difficulty, as well as the comorbidity between internalising and externalising disorders, had an impact on parent–child agreement. We hypothesised that parent–child agreement would be higher when reporting the child’s conduct and emotional problems in children presenting with only internalising or comorbid externalising and internalising difficulties, than for children presenting with only externalising problems. This was based on previous literature [16] that demonstrated higher agreement for children diagnosed with depression and anxiety, than conduct problems. This was explored as two separate hypotheses: one for agreement on reports of conduct problems, and one for agreement of reports on emotional problems.
2. The second aim was to examine the effect of gender and age on parent–child agreement. With regards to this no specific hypothesis is stated, as findings from previous literature have been mixed and inconclusive and we aimed to clarify this literature using a large clinical sample.

## Methods

### Sample of clinical records

This project involved the use of a large dataset of clinical records provided by the Child Outcomes Research Consortium (CORC), a collaboration that collects routine outcome data from multiple informants, in more than 70 CAMH services across the UK [2]. In line with ethical research frameworks, all data provided was anonymized, maintaining the confidentiality of both CORC member services and individual service users. The final sample included 16,754 clinical records of treatment episodes for children from 11 to 17 years old, seen in the time period between 1998 and 2013. These records were obtained from the assessment stage when the outcome measures were administered for the first time with each child. Of these, 9518 (57%) were female, with mean age 14.3 (SD = 1.67) and 7184 (43%) were male, with mean age 13.6 (SD = 1.75). Additionally, the majority of these were White (76%), followed by 6% from Asian/Asian British background, 4% Black/Black British, 4% from a mixed background and 4% from other ethnic backgrounds.

### Measures

#### Clinician-reported presenting problems

Clinicians completed a form rating twelve presenting problems for each child at the assessment stage. Ratings are based on the clinical judgement of individual clinicians and do not need to imply a diagnosis. The twelve presenting problems included in the form were: hyperkinetic, emotional, conduct, eating, psychosis, deliberate self-harm, autism spectrum disorder, learning disability, developmental, habit, substance misuse and other problems. The clinician was asked to provide ‘yes’ or ‘no’ answers, as to whether each of these problems was present for a child. The most common presenting difficulties reported in this sample were emotional (57%) and conduct problems (15%).

These clinician-reported presenting problem variables were used to divide the sample into seven groups based on the children’s presenting difficulties (see Table 1). The first three categories represent the main groups of interest to this study: those identified as having only externalising problems (EXT), those with only internalising problems (INT), and those identified as having both externalising and internalising problems (COM) but none of the other difficulties. The remaining four were comparison groups, to explore the influence of other combinations of presenting difficulties on agreement: those with externalising and other problems (EXT and OTHER), internalising and other (INT and OTHER), externalising internalising and other (COM and OTHER) and any other problem (OTHER).

**Table 1:** Demographic information for the children in each problem group

Group	Presenting problems^a^	n (%)	Mean age (SD)	Males %
EXT	Conduct	1345 (8)	13.41 (1.54)	65
INT	Emotional	6373 (38)	14.15 (1.77)	36
COM	Conduct and emotional, excluding other problems	508 (3)	13.47 (1.61)	57
EXT and OTHER	Conduct and any other, excluding emotional	421 (3)	13.45 (1.60)	72
INT and OTHER	Emotional and any other, excluding conduct	2317 (14)	14.30 (1.73)	34
COM and OTHER	Conduct, emotional and any other	306 (2)	13.84 (1.63)	51
OTHER	Any other, excluding conduct and emotional	5484 (33)	13.87 (1.74)	46
Total	–	16,754 (100)	13.98 (1.75)	43

^a^ ‘Other’ presenting problems include: hyperkinetic, eating, psychosis, deliberate self-harm, autism spectrum disorder, learning disability, developmental habit, substance misuse and other

#### Strengths and Difficulties Questionnaire (SDQ)

The SDQ is a short questionnaire of 25 items used to assess the positive and negative behaviours of children and indicate the extent of their difficulties [6, 39]. The SDQ contains five subscales with 5 items each, representing different behavioural, social and emotional domains. These include conduct problems, emotional problems, hyperactivity, peer problems and prosocial behaviour. Scores ranging from 0 (no difficulties) to 10 (severe difficulties) are generated for each individual scale to indicate the extent of the child’s difficulties for each domain. In terms of outcome information, the main variables used for this study were the scores from the conduct and emotional scales of the SDQ, for both parents and children. Data were collected using the self-report version, which was developed for young people between the ages of 11–17 [6] and the parent-rated version aimed to be completed by parents/carers of children aged 4–17 [39–41].

Findings concerning the validity of the parent version of the SDQ indicated that it operated equally well as other well-established measures, such as the Rutter questionnaires or the Child Behaviour Checklist [39, 42]. It also demonstrated adequate criterion validity in relation to clinical diagnosis, as a correlation of .47 was found between the total difficulties score and diagnostic interview features [43]. Moreover, Goodman and colleagues [6] found satisfactory internal consistency for the self-report version of the SDQ in an adolescent population (emotional scale a = .75; conduct scale a = .72) and also confirmed that the self-report version could be used effectively to distinguish between children in a clinical sample from those in a community sample (concurrent validity = .82).

### Procedure

#### Exclusion criteria

**Fig. 1:**
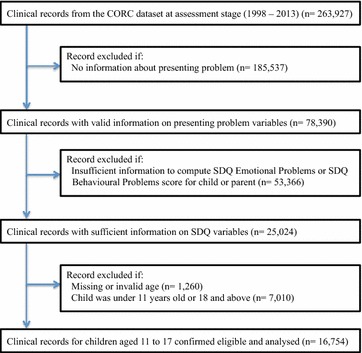
Flow chart demonstrating the sample selection process

The initial dataset provided by CORC contained 263,927 clinical records. However, large amounts of essential data (e.g. presenting problems) were missing, necessitating sample selection based on the following three main exclusion criteria: (1) records with no information about clinician-reported presenting problems for the child, as these formed the basis for dividing the sample into groups; (2) records with insufficient information to compute SDQ Emotional Problems or SDQ Behavioural Problems score for children or parents, as the main premise of this project was to investigate the reporting behaviours of children and parents; (3) records of young people under the age of 11 or over 17, in accordance to SDQ guidelines about the age suitability of the self-report version [6]. Figure 1 demonstrates a flowchart of the selection process. Sample selection was closely monitored, by comparing the descriptive statistics and distributions of the main variables of interest (such as the SDQ conduct and emotional scale scores) before and after the sample selection, and analyses indicated that the selection process did not change the data significantly or introduced bias in the distribution of key variables.

#### Data analysis

Before conducting the analysis, it was important to acknowledge the possible influence of missing values on the results. Based on the sample inclusion criteria, all the children had self-report data but not all had parent data. Effectively, this meant that records with missing parent scores would not be included in the correlation analysis. In order to identify whether these missing values would create a bias in the sample, the proportion of parents and children who both completed the SDQ was investigated for each age. It was found that the older the children were, the smaller the percentage was of those who had both child and parent reports. The distributions of the child scores on both SDQ scales for those with only child reports were found to be similar to the distributions of those who had both child and parent reports, thus it was decided to conduct the analysis on the latter group.

In order to test whether parent–child agreement on the SDQ conduct and emotional scales varied by the child’s presenting problem, the data was analysed using Pearson’s r correlations.^1^ Following that, a test of multiple independent correlations [44] was conducted for each scale, in order to identify whether the aforementioned group coefficients significantly differed from each other, thus representing a real difference in the population. Then, Fisher’s Z transformations [45] were used to investigate whether agreement differed between groups when reporting on the problems that defined the child’s presentation; for example, by comparing the INT group agreement on the emotional scale with the EXT group agreement on the conduct scale. Lastly, pairwise tests of correlated correlations [46] were run to test the difference between the coefficients of the conduct scale and those of the emotional scale for each problem group. Pearson’s r correlations were finally conducted to explore agreement for different ages and gender.

All in all, this study employs ten statistical hypothesis tests. For each test, we report the uncorrected p value. This is recommended for research situations such as ours, where tests are used to investigate specific hypotheses developed prior to seeing the data [47, 48]. For the exploratory analyses relating to the second research aim, we do not employ significance tests, but report confidence intervals to indicate the uncertainty around the observed correlations.

## Results

### Description of parent and child SDQ scores

The descriptive statistics for each problem group were investigated for the child and parent conduct and emotional scales of the SDQ (see Table 2), and their distributions were found to be approximately normal. It appears that patterns in mean scores were similar for children and parents for both scales, and they both reported problems that were relevant to the child’s presentation as stated by the clinician. For example, mean scores on the conduct scale were higher for groups that included externalising problems (EXT, COM, EXT and OTHER, COM and OTHER), while mean scores on the emotional scale were higher for groups including internalising problems (INT, COM, INT and OTHER, COM and OTHER) compared to those that did not. Additionally, parents tended to have higher means than children for almost all groups on both SDQ scales, with the exception of the INT and INT and OTHER groups on the conduct scale scores.

**Table 2:** Group descriptive statistics for parent/child scores on the SDQ emotional and conduct scales

Group	Emotional scale SDQ				Conduct scale SDQ			
	Child scores		Parent scores		Child scores		Parent scores	
	Mean (SD)	95% CI	Mean (SD)	95% CI	Mean (SD)	95% CI	Mean (SD)	95% CI
EXT	4.34 (2.70)	[4.19, 4.48]	4.88 (2.72)	[4.72, 5.23]	4.82 (2.17)	[4.71, 4.94]	5.41 (2.42)	[5.27, 5.54]
INT	5.98 (2.58)	[5.91, 6.04]	6.22 (2.64)	[6.15, 6.29]	3.18 (2.12)	[3.13, 3.23]	3.08 (2.35)	[3.01, 3.14]
COM	4.66 (2.59)	[4.44, 4.89]	5.29 (2.65)	[5.04, 5.53]	4.47 (2.18)	[4.55, 4.93]	5.29 (2.42)	[5.06, 5.51]
EXT and OTHER	4.43 (2.63)	[4.18, 4.68]	5.28 (2.61)	[5.01, 5.55]	5.34 (2.31)	[5.11, 5.56]	6.09 (2.49)	[5.84, 6.35]
INT and OTHER	6.21 (2.54)	[6.10, 6.31]	6.23 (2.63)	[6.12, 6.35]	3.49 (2.16)	[3.41, 3.58]	3.37 (2.39)	[3.26, 3.47]
COM and OTHER	5.12 (2.62)	[4.83, 5.42]	5.79 (2.73)	[5.45, 6.14]	4.97 (2.30)	[4.72, 5.23]	5.77 (2.55)	[5.45, 6.08]
OTHER	5.35 (2.70)	[5.28, 5.43]	5.55 (2.76)	[5.47, 5.63]	3.69 (2.31)	[3.63, 3.75]	3.72 (2.60)	[3.65, 3.80]

*CI* confidence interval

### Parent–child agreement by presenting problem

#### Correlations for the conduct scale

The first hypothesis postulated that there would be higher parent–child agreement on reports of conduct problems, for children in the INT and COM groups, than those in the EXT group. Therefore, a correlation was run for each problem group to indicate the agreement between the children’s and parents’ scores on the conduct dimension of the SDQ. As can be seen in Table 3, parent and child scores were positively correlated for all problem groups, with the OTHER group having the highest correlation, followed closely by the INT group.

**Table 3:** Correlations between parent–child scores on the conduct and emotional scales, for each problem group

Group	Conduct scale			Emotional scale		
	n	r	95% CI	n	r	95% CI
EXT	1219	.50	[.45, .54]	1214	.56	[.52, .60]
INT	5168	.62	[.60, .64]	5160	.54	[.52, .57]
COM	447	.51	[.44, .57]	446	.49	[.41, .56]
EXT and OTHER	368	.53	[.45, .61]	366	.56	[.47, .63]
INT and OTHER	1987	.61	[.58, .65]	1983	.50	[.46, .54]
COM and OTHER	247	.49	[.38, .58]	246	.57	[.48, .64]
OTHER	4688	.64	[.61, .65]	4682	.55	[.52, .57]

*CI* confidence interval

The test of multiple independent correlations indicated that within the conduct scale, at least some of the correlations significantly differed between the groups (C(α) = 64.4, df = 6, p < .0001). More specifically, the correlation coefficients indicated that parents and children in problem groups that excluded externalising problems (INT, INT & OTHER, OTHER) seemed to agree more on conduct scores than groups that included externalising (EXT, COM, EXT and OTHER, COM and OTHER). Note in particular that the COM and COM and OTHER groups had smaller correlation coefficients than groups that did not include externalising difficulties, despite having comorbid internalising problems. These findings partly support our hypothesis, as they demonstrate that internalising groups have better parent–child agreement than the externalising ones whilst reporting conduct problems. However, it seems that it is the absence of externalising, rather than the presence of internalising difficulties that relates to higher parent–child agreement.

#### Correlations for the emotional scale

The second set of correlations was comparing child and parent scores on the emotional dimension of the SDQ. For the hypothesis to be supported it was again expected that correlations would be higher for groups with internalising problems and particularly the COM group, than the group with only externalising difficulties. As can be seen in Table 3, parent and child scores were positively correlated for all problem groups, with the COM and OTHER problem group having the highest correlation, while the COM group had the lowest correlation. Overall, it appeared that groups including internalising problems tended to have slightly lower parent–child agreement (INT, COM, INT and OTHER), with the exception of the COM and OTHER. These results do not support the initial hypothesis; rather, if anything the opposite tended to occur, that groups with externalising difficulties showed greater agreement on scores of emotional difficulties. However, the test of multiple independent correlations found no significant difference between any of the group correlations for the emotional scale (C(α) = 8.6, df = 6, p = .198). Therefore, there was no evidence that the presenting difficulties of the child affect the level of parent–child agreement on the emotional scale.

#### Comparing agreement between scales

Results from some groups within each scale seemed to demonstrate a paradoxical pattern (see Table 3); parents and children agreed more on problems that were not considered to be part of their presenting difficulties. Fisher’s Z transformations indicated that the difference between groups, when reporting on the problems that defined their presentation, was significant (z = −2.07, p = .039), but quite small. Those in the INT group showed slightly higher agreement when reporting emotional difficulties (r = .544) than those in the EXT group when reporting on conduct difficulties (r = .496). The pairwise tests of correlated correlations (see Table 4) indicated that for the four groups which included externalising problems there was either no or weak evidence of a difference between the correlations on the conduct and the emotional scale. In the three groups that excluded externalising problems, the difference between the correlations was significant and indicated that it was higher for the conduct scale than the emotional one.

**Table 4:** Pairwise tests of correlations of the SDQ conduct and emotional scales for each group

Group	Z value	p
EXT	2.10	.035
INT	−6.31	<.001
COM	−.25	.805
EXT and OTHER	.53	.598
INT and OTHER	−5.13	<.001
COM and OTHER	1.20	.228
OTHER	−6.73	<.001

### Parent–child agreement by age

Further correlations were conducted to explore whether parent–child agreement on the two scales varied as a result of the child’s age. As can be seen in Table 5, all correlations were moderately strong for both scales and for all age groups. For the conduct scale, the highest agreement was found for age 11 (r = .662) and the lowest for age 14 (r = .615), while for the emotional dimension the highest was for age 12 (r = .589) and the lowest for age 16 (r = .513). Overall, there was a slight indication that parent–child agreement varied with age and that younger ages were associated with higher agreement, especially when reporting emotional difficulties. For the conduct scale the differences in agreement between ages tended to be smaller. Additionally, it appeared that independently of age, children and parents tended to agree more on conduct difficulties rather than emotional.

**Table 5:** Correlation coefficients between parent–child scores on the conduct and emotional scales, for each age group

Age	Conduct scale			Emotional scale		
	n	r	95% CI	n	r	95% CI
11	1487	.66	[.62, .69]	1485	.57	[.53, .62]
12	2111	.65	[.60, .67]	2105	.59	[.57, .64]
13	2421	.62	[.59, .65]	2421	.57	[.52, .58]
14	2796	.62	[.59, .65]	2783	.58	[.55, .61]
15	2811	.64	[.61, .67]	2801	.53	[.50, .57]
16	1726	.63	[.57, .65]	1727	.51	[.47, .56]
17	772	.62	[.53, .63]	775	.52	[.47, .59]

*CI* confidence interval

### Parent–child agreement by gender

Lastly, we investigated whether parent–child agreement on the conduct and emotional scales varied as a result of the child’s gender (see Table 6). Correlations were moderate in size, for both males and females, on both scales. Within scales, parent–child agreement for males was very similar to that of females. Between scales, both genders tended to show greater agreement with parents on the conduct scale compared to the emotional. Overall, the findings indicated that gender does not seem to have an effect on parent–child agreement on reports of either conduct or emotional difficulties.

**Table 6:** Correlations between parent–child scores on the conduct and emotional scales, for males and females

Gender	Conduct scale			Emotional scale		
	n	r	95% CI	n	r	95% CI
Male	6276	.63	[.61, .65]	6264	.54	[.54, .58]
Female	7799	.64	[.61, .64]	7784	.53	[.53, .57]

*CI* confidence interval

## Discussion

The current study investigated parent–child agreement on ratings of the child’s conduct and emotional problems and whether this varied as a result of the child’s presenting difficulties, age and gender. It was firstly hypothesised that there would be higher parent–child agreement when rating the child’s conduct problems, for children whose presentation included internalising or both internalising and externalising difficulties, than those presenting with only externalising problems. The findings partly supported this hypothesis, as it was revealed that those in groups including internalising presentations had higher agreement compared to those with externalising presentations, which is in line with previous findings [16]. Nevertheless, parent–child agreement in those presenting with both internalising and externalising problems was very similar to groups including externalising difficulties. Thus, the hypothesis could not be fully supported and it appeared that the difference in agreement was the result of the absence of externalising difficulties, rather than the presence of internalising. As the aim of this study was to investigate the relationship between presenting problem and agreement, it would be of interest for future studies to explore the underlying processes that might be guiding this variation. For example, one possible explanation for the findings could be that in this sample perceptual biases resulting from the child’s presenting difficulty reduced their ability to assess their own problematic symptomatology [20, 21], which led to them underreporting their difficulties. This is in accordance to previous literature, which postulated that externalising difficulties in childhood have been associated with positively biased perceptions regarding one’s self [22–24, 49].

Another explanation for the aforementioned finding could be that comorbidity is often associated with higher levels of dysfunction and difficulty [31]. Therefore, lower than expected agreement on reports for children presenting with both externalising and internalising difficulties may have been a result of the severity of the child’s presentation, which further impacted on their ability to report their own behaviours.

Secondly, it was hypothesised that parent–child agreement would be higher when reporting the child’s emotional difficulties, for children presenting with either comorbidity of externalising and internalising, or with only internalising difficulties, as opposed to those with only externalising problems. The hypothesis was not supported by the results, as it appears that the type of difficulty the child is presenting with may not have a large impact on agreement when rating the child’s emotional difficulties.

Moreover, the mean ratings on the emotional scale indicated that parent ratings in all problem groups were slightly higher than the children’s ratings. This is consistent with Herjanic and Reich (1997) who found that in a clinical sample parents reported greater emotional problems for their children than the children themselves [19]. Thus, even though child difficulties in the current study do not seem to correlate to the degree of disagreement for emotional problems, a disagreement still exists. One possible explanation could be that other factors may have a greater influence on parent–child agreement on emotional problems than the child’s presenting difficulties; for example, parent characteristics [50]. These findings are contradictory to other studies which have proposed that parents often under-report their children’s emotional difficulties, as these are less observable and more subjective than externalising symptoms [19, 28, 36]. However, this contradiction might be due to studies using community based samples, whereas the current study focused on clinical samples whereby parents and children might be more aware of the problems given they are attending mental health services. Additionally, results indicated that parent–child agreement was overall moderate-to-high for both scales of the SDQ. It is possible that agreement between reporters is higher using the SDQ as the children and parents respond on exactly the same constructs, compared to if different measures of psychopathology had been used for parent and child reporters.

This study also explored the effect of age and gender on parent–child agreement. With regard to age, there was some indication that parent–child agreement was higher in younger adolescents than in older adolescents, especially in relation to reports on emotional difficulties, a finding that has been reported in some existing literature [3, 51, 52]. One explanation could be that younger children disclose their difficulties to parents more often and they also spend more time with them, which may allow parents to recognise difficulties [3]. For the present investigation, however, the differences in agreement for different ages did not appear to be large, which is consistent with other studies that have not found the child’s age to have a great impact on parent–child agreement [37, 53]. Given the large sample used in this study, we were able to estimate correlations with a high degree of precision. However, there may be a risk of bias in the age comparisons due to a selection effect, since older children were less likely to have a parent rating compared to younger children.

Parent–child agreement did not seem to vary as a function of gender, for both the conduct and emotional scales, a finding in line with previous studies [3, 37]. However, there have been some studies which demonstrated some gender difference in parent–child agreement [38, 53], albeit these studies have mixed findings. It is possible that parent gender might interact with child gender in predicting extent of parent–child agreement, for instance, Jensen and colleagues [54] reported higher agreement between mothers and sons compared with fathers and sons when reporting behavioural difficulties [54]. Given we did not have information regarding the reporting parent’s gender, this interaction effect could not be investigated.

Lastly, a paradoxical result was obtained when investigating agreement within scales. Parents and children seemed to agree more on problems that did not define their presentation as assigned by the clinician. For instance, within the conduct scale, agreement was higher for groups without externalising difficulties, whereas within the emotional scale agreement was higher for those presenting with externalising problems. One possibility could be that having a particular presenting problem increases biased perceptions regarding that problem’s symptoms, but not the awareness of their functioning in other areas. For example, having externalising difficulties may hinder the awareness of one’s conduct problems, but not the awareness of emotional functioning. Another explanation could be that either some of the parents or some of the children do not in fact agree with the clinician’s judgement of what the child’s presenting difficulties are. This emphasizes even further the need of exploring discrepancies and disagreements not only between informants, but with clinician reports as well.

### Strengths and limitations

This study represents one of the largest investigations of parent–child agreement in a clinical sample. Utilising clinicians’ reports in essence makes this a study of parent–child agreement in the context of clinical assessment of presenting problems, which represents a real strength. However, the use of clinician judgement as the key grouping variable might also represent a limitation, because clinician judgment and decision making, similarly to all other reporters, is subject to biases and imperfect reliability [55–57]. Since ours is a real-world setting, the type and thoroughness of assessment, as well as the timing of completion of the presenting difficulties form, may have varied between services and practitioners. Clinician ratings may have been based on parents’ and children’s reports to varying degree, so the problem ratings and the parents’ and children’s SDQ scores cannot be regarded as independent sources of information.

We could not examine other factors that might impact on agreement, such as parent characteristics and perspectives, or contextual influences [50, 58] as these data were not available as part of this service dataset. Furthermore, the SDQ does not measure some problems encountered by the age group studied here. In particular, self-harm, psychosis, and eating disorders are not measured by the SDQ. This meant that parent–child agreement on what, for some children, may have been the main problem, could not be assessed.

### Implications and conclusions

The findings of the current study indicate that parent–child agreement did vary as a result of the child’s presenting difficulties for reports of the child’s conduct problems, but not on reports of emotional difficulties. More specifically, it was found that the absence of externalising difficulties was associated with greater parent–child agreement on conduct problems. Lastly, children and parents seemed to agree more on problems that did not relate to the presenting difficulties assigned by the clinician. It would be useful for future studies to investigate further why informant discrepancies are more pronounced for certain difficulties than others. Longitudinal investigations in particular might help shed light on how parent–child agreement may change as a result of the child receiving treatment or as a result of changes in the child’s presentation. More specifically, the trajectories related to comorbidity may be of interest; for instance, whether the impact that one disorder exerts on self-awareness changes as a result of the development of another difficulty and how that influences self-awareness. Ethnicity, religion and other societal influences would also be important to explore, as these can have an impact on parent–child agreement and might provide valuable information when analysing data from multicultural societies [59]. Lastly, a further investigation of agreement variation between children or parents with other informants such as teachers would be beneficial.

Findings from this study indicate that discrepancies between parents and children can provide meaningful information and should not be used to justify the use of a single informant. More specifically in clinical practice, the investigation of the factors related to these discrepancies may provide relevant information to guide the assessment and treatment processes. Collecting information from multiple informants should remain a priority in CAMHS, with the aim to better integrate such information by identifying the common elements, while at the same time preserving the individuality of each report to provide an insight into the informant’s perspective and level of awareness of his/her own difficulties [1]. The meaningful interpretation of informant discrepancies could also be useful for better understanding and critically assessing research outcomes and reaching conclusions from empirical work [15, 60].

## Abbreviations

ADHD: Attention Deficit Hyperactivity Disorder
CAMHS: Children and Adolescent Mental Health Service
COM: comorbid externalising and internalising problems
COM and OTHER: comorbid externalising and internalising problems and any other problem
CORC: Child Outcomes Research Consortium
EXT: externalising problems
EXT and OTHER: externalising problems and any other problem
INT: internalising problems
INT and OTHER: internalising problems and any other problem
OTHER: any other problem
SDQ: Strengths and Difficulties Questionnaire

## Footnotes

1 Concordance correlation coefficients (CCC) [61] and intraclass correlation coefficients (ICCs) between parents and children were also conducted as sensitivity analyses on both the conduct and emotional SDQ scales, to test whether these would be different to the Pearson correlations (PC). The results indicated that there was very little difference between the three (max. difference was .03), thus the use of PC was justified, as the results would not substantially change if CCC or ICC were used.
